# Internal Astigmatism and Its Role in the Growth of Axial Length in School-Age Children

**DOI:** 10.1155/2018/1686045

**Published:** 2018-04-18

**Authors:** Liangcheng Wu, Chenghai Weng, Fei Xia, Xiaoying Wang, Xingtao Zhou

**Affiliations:** ^1^Department of Ophthalmology, Jing'an District Centre Hospital of Shanghai, Fudan University, Shanghai, China; ^2^Department of Ophthalmology and Vision Science, Eye and ENT Hospital, Shanghai Medical College, Fudan University, Shanghai, China

## Abstract

**Objectives:**

To explore the role of internal astigmatism (IA) in the growth of axial length (AL) in school-age children.

**Methods:**

Total astigmatism (TA), corneal astigmatism (CA), and AL of all children in Jing'an District 2nd Centre Primary School in Shanghai were measured. In IA, the difference between TA and CA was also calculated using vector analysis. The association of axial length with IA, genders, and age was analyzed using linear regression. The difference of IA between both eyes was also calculated. The AL between both eyes was compared using paired samples *t*-test when DIA = 0 D, <0.5 D, and ≥0.5 D.

**Results:**

Six hundred and twelve cases (98.23%) in 623 children aged 7–12 yrs older entered into the study. Genders, age, and IA all affected AL. This could be represented by a linear regression line in the form AL = 21.46 − 0.43∗gender + 0.22∗age + 0.46∗IA (male = 1, female = 2; *t* = 7.01, *P* < 0.01 for sex; *t* = 11.6, *P* < 0.01 for age; and *t* = 6.6, *P* < 0.01 for IA; *R*^2^ = 0.16). The AL in the eye with larger IA was also longer when DIA was larger than 0.5 D (*t* = 2.65, *P* < 0.01).

**Conclusions:**

IA was observed to be associated with AL and might be a risk factor of the onset and progress of myopia in school-age children.

## 1. Introduction

Myopia is becoming a common public health problem worldwide especially in eastern Asian countries [1]. The myopia-related complications are major causes of visual impairments and blindness [1–3]. To explore the mechanism and risk factors of myopia is always the hot focus of myopia study. It is well known but still under debate that astigmatism takes an important role in the creation and progress of myopia in the growth and development stage of humans and some animals. Many studies supported that astigmatism was a risk factor for myopia progress or creation [4–6]. But not all studies have shown an association between the presence of astigmatism and the progression of myopic refractive errors. Czepita and Filipiak reported that a negative correlation was found between cornea astigmatism (CA) and myopia progress, and a positive correlation was observed between TA and myopia [7]. Astigmatism or total astigmatism (TA) consists of CA and internal astigmatism (IA). TA and CA can be independently measured. The difference between the two, internal astigmatism (IA), is thought to arise from the internal optics of the eye, including asymmetries related to the crystalline lens [8]. We speculate that IA may play a more important role in the progress or creation of myopia. In the present study, we examined TA, CA, and axial length (AL) for all children in Jing'an District 2nd Centre Primary School in Shanghai, and the purpose was to explore the role of IA in the growth of eye axial length.

## 2. Methods

The study was based on a school survey and was approved by the Jing'an District Hospital Ethics Committee and conducted in accordance with the principles of the Declaration of Helsinki. The study was a part of routine school refraction screening program and was performed in Jing'an District 2nd Centre Primary School. Jing'an District 2nd Central Primary School was a school with 5 grades, 20 classes, and 623 children including 335 male and 288 females in 2016. All children were asked to test for uncorrected visual acuity, presenting visual acuity, refraction, and axial length. The eyeball axial length was measured by a commercial instrument (IOLMaster; Carl Zeiss Meditec AG, Jena, Germany). Noncycloplegic autorefraction was performed using an Auto Ref-Keratometer (HRK-7000A; HUVITZ Co. Ltd., Korea). In fully automated mode, the Auto Ref-Keratometer performed at least five autorefractions in each eye and gave a standardized value as its output.

TA, CA, and IA were expressed in negative correcting cylinder form and were described at the corneal vertex surface. So the vertex distance was set as zero during autorefractor examination and TA was given in the autorefractor output. CA axis was set along the *K*_min_ meridian. We calculated CA based on the autokeratometry reading using a corneal refractive index of 1.3375. This takes into account the negative refractive power of the posterior corneal surface [8]. CA was calculated as *K*_min_ − *K*_max_, where *K*_min_ represents the meridian with the least refractive power and *K*_max_ the meridian with the greatest refractive power. IA was calculated as the vector difference between TA and CA and was detailed described in other studies [8, 9].

The difference of IA between both eyes (DIA) was also calculated; these children were divided into 3 groups according to the DIA including DIA = 0 D, DIA <0.5 D, and DIA ≥ 0.5 D.

### 2.1. Statistical Analysis

Statistical analysis was performed using SPSS software version 13 (SPSS Inc., Chicago, IL). IA and DIA were expressed as absolute value (positive value) in statistical process. The age, AL, and IA in between both genders were compared using independent-samples *t*-test. Paired-samples *t*-test was used to compare AL between both eyes when DIA = 0 D, DIA < 0.5 D, and DIA ≥ 0.5 D. Linear regression was used to analyze relationship between AL and age, relationship between IA and age, relationship between AL and IA, and relationship between AL and some factors including age and genders along with IA. The sex ratio between boys and girls in various ages was compared using a chi-square test. *P* < 0.05 was considered as statistically significant.

## 3. Results

**Figure 1:**
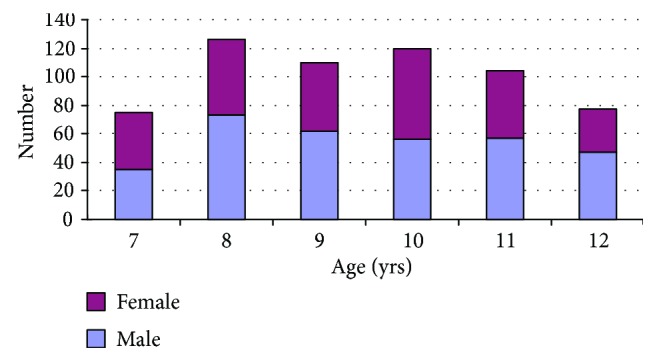
The distribution of gender at various age in 623 children.

Six hundred and twelve cases (98.23%) in 623 children aged 7–12 yrs older were enrolled into the study, including 330 boys and 282 girls. Four children were excluded from the study because of being absent from school and 7 because of obtaining unreliable data. The distribution of age in both genders was showed in Figure 1; the average age between genders was not significantly different (independent-samples *t*-test: 9.51 ± 1.59 yrs for boys versus 9.41 ± 1.56 yrs for girls, *t* = 0.79, and *P* > 0.05), and there was no significant difference in sex ratio between boys and girls in all ages (chi-square test: χ^2^ = 6.81, *P* > 0.05). The refractive errors including sphere and TA, CA, and AL along with DIA were showed in Table 1.

### 3.1. Association of AL with IA, Age, and Gender

**Figure 2:**
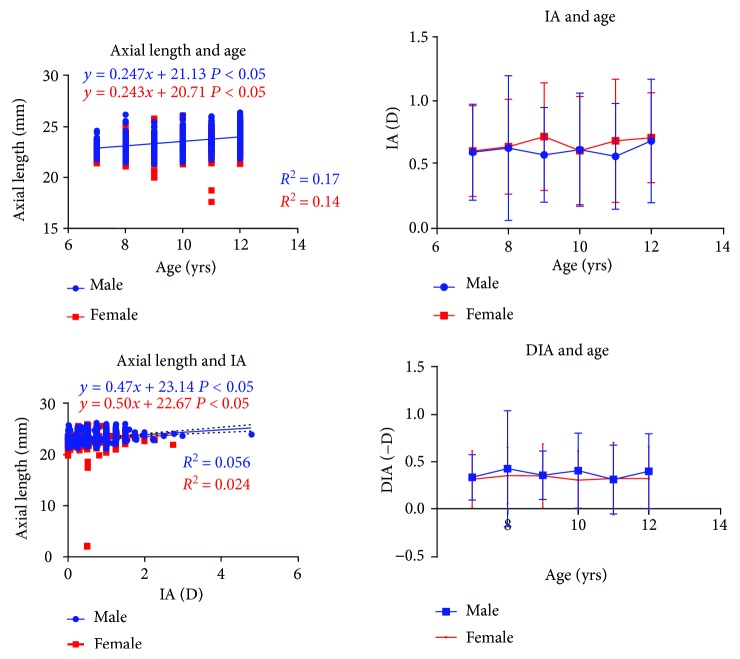
The relationship between axial length and age as well as the relationship between internal astigmatism age.

The AL of boys was 23.46 ± 0.92 mm, and that of girls was 23.01 ± 0.99 mm. The AL of boys was longer significantly than that of girls (independent-samples *t*-test: *t* = 6.57, *P* < 0.001). There was a linear relationship between AL and age in both genders (linear regression analysis: *t* = 10.56, *P* < 0.001 in male; *t* = 9.14, *P* < 0.001 in female; Figure 2).

The IA was 0.61 ± 0.46(−D) in boys and 0.66 ± 0.41(−D) in girls; there was no significant difference between genders (independent-samples *t*-test: *t* = 1.88, *P* > 0.05). IA maintained stable with age in both genders (linear regression analysis: *t* = 0.56, *P* > 0.05 in male; *t* = 0.053, *P* > 0.05 in female). But a linear relationship was found between AL and IA (linear regression analysis: *t* = 6.27, *P* < 0.001 in male; *t* = 3.68, *P* < 0.001 in female; Figure 2).

Linear regression analysis showed that genders, age, and IA all affected AL. This could be represented by a linear regression line in the form AL = 21.46 − 0.43∗gender + 0.22∗age + 0.46∗IA (male = 1, female = 2; *t* = 7.01, *P* < 0.01 for sex; *t* = 11.6, *P* < 0.01 for age; and *t* = 6.6, *P* < 0.01 for IA; *R*^2^ = 0.16).

### 3.2. Association of AL with DIA

The DIA was −0.38 ± 0.42(D) in male and −0.33 ± 0.33(D) in female; the difference was not significant between genders (independent-samples *t*-test: *t* = 1.62, *P* > 0.05) and also did not vary with age (one-way ANOVA: *F* = 0.66, *P* > 0.05 in male; *F* = 0.18, *P* > 0.05 in female), but when DIA was larger than 0.5 D, the axial length with larger IA was also longer (pared-samples *t*-test: *t* = 2.65, *P* < 0.01) and was showed in Table 2.

## 4. Discussion

Cycloplegic refraction was a need in school-age children due to high accommodation. However, cycloplegic refraction was difficult to perform in school. AL is the primary determinant of nonsyndromic myopia. It is a parameter representing the combination of anterior chamber depth, lens thickness, and vitreous chamber depth of the eye. The AL elongation in children can be related to the normal growth of the eyeball and, thus, affect the refractive status of the eye. Considering the contribution of AL, lens power, and corneal power together using multiple linear regression analyses, AL can explain up to 96% of the variation of refraction in populations [10]. The AL is a valid parameter for monitoring myopic progression [10, 11]. In our study, the AL was used to evaluate the refractive status and the growth of the eyeball in school-age children.

Our study showed that genders, age, and IA all affected the AL. A linear relationship was found between AL and IA. In fact, many factors including age, genders, outdoor activities, ethics, refraction correction pattern, and so on all affect the AL growth. In order to exclude these disturbing factors, we compared the AL difference between both eyes when DIA = 0 D, 0–0.5 D, and ≥0.5 D. The eye with larger IA had also a longer AL (*P* < 0.05) when the DIA was more than 0.5 D, which suggested that IA might be a stimulus factor of myopia progress or occurrence.

One study showed that it was possible that astigmatism was a passive byproduct of abnormal posterior axial eye growth [12]. They pointed out that axial eye growth might alter anterior ocular structures through stretching and the fact that changes in axial length correlated significantly with changes in corneal power or lens power during early infancy. Although these results seem to be contradictory, we still speculate that IA was a stimulus factor of AL growth rather than the outcome of AL elongation. Firstly, IA was independent from age, which meant that AL growth with age did not increase IA. Secondly, the AL growth mainly led to increase of CA [12]. Thirdly, the conclusion from the animal experiment was based on eyeball abnormal growth [12], and in the present study, most patients were less than 26 mm.

Blurred image focusing on the retina was considered the mechanism of myopia occurrence or progress. Deprivation of a focused retinal image can cause high myopia in primates and chicks, and peripheral retinal hyperopic defocus imposing peripheral hyperopic defocus produces axial myopia [13, 14]. Even with spectacle corrections, IA can create a blurred retinal image especially in the peripheral retina because there is an axial distance between the corrected spectacle and the intraocular lens (the internal astigmatism location). The study confirmed the linear relationship between AL growth and IA. It is this line of reasoning, along with reports of an association between astigmatism and the onset of myopia in animal experiment [4].

We acknowledge certain limitations in our study. Firstly, the present study had a cross-sectional design based on a single elementary school. The conclusion should be applied with caution and need to be further studied in the future. Secondly, AL was used as the only parameter for monitoring myopia progress in the study. In fact, other ocular structures such as the cornea, aqueous humor, lens, and the vitreous humor also contribute to the refractive status of a given human eye. The ratio between the AL and corneal radius may be a better indicator of myopia [15]. Thirdly, our study does not analyze the relationship between AL and TA (CA) and the relationship between high myopia and high astigmatism. Finally, in the corneal refractive astigmatism calculated from autoref, the results were rather coarse; it is better to use some advanced machine to measure corneal astigmatism, such as IOL master [16].

In conclusion, IA was observed to be associated with axial length and might be a risk factor of the onset and progress of myopia in school-age children.

## Figures and Tables

**Table 1:** Axial length, IA, and DIA in school children in Jing'an District 2nd Primary School 2016.

	Axial length (mm)		Sphere (−D)		TA (−D)		CA (−D)		IA (−D)		DIA (−D)	
Age (yrs)	Male	Female	Male	Female	Male	Female	Male	Female	Male	Female	Male	Female
7	22.93 ± 0.56	22.56 ± 0.58	0.16 ± 0.36	0.29 ± 1.13	0.91 ± 0.93	0.6 ± 0.44	0.92 ± 0.89	0.6 ± 0.52	0.60 ± 0.37	0.61 ± 0.36	0.34 ± 0.24	0.31 ± 0.30
8	23.15 ± 0.72	22.60 ± 0.78	0.31 ± 0.77	0.41 ± 0.97	0.89 ± 0.75	0.85 ± 0.56	0.84 ± 0.70	0.85 ± 0.62	0.63 ± 0.57	0.64 ± 0.37	0.43 ± 0.62	0.35 ± 0.30
9	23.41 ± 0.84	22.94 ± 0.93	0.37 ± 0.65	0.64 ± 1.29	0.79 ± 0.62	0.84 ± 0.75	0.83 ± 0.67	0.84 ± 0.74	0.58 ± 0.37	0.72 ± 0.42	0.36 ± 0.26	0.35 ± 0.34
10	23.37 ± 0.77	23.08 ± 0.93	0.51 ± 0.97	0.78 ± 1.30	0.85 ± 0.63	0.76 ± 0.58	0.88 ± 0.65	0.75 ± 0.60	0.63 ± 0.45	0.61 ± 0.43	0.41 ± 0.40	0.31 ± 0.39
11	23.71 ± 1.02	23.26 ± 1.19	0.97 ± 1.31	1.11 ± 1.40	0.89 ± 0.72	1.04 ± 0.85	0.88 ± 0.74	1.02 ± 0.83	0.57 ± 0.42	0.69 ± 0.48	0.31 ± 0.36	0.32 ± 0.39
12	24.16 ± 1.06	23.78 ± 1.06	1.22 ± 1.37	1.67 ± 1.65	1.05 ± 0.79	0.90 ± 0.49	1.10 ± 0.84	0.84 ± 0.51	0.68 ± 0.49	0.71 ± 0.35	0.40 ± 0.40	0.32 ± 0.34
Average	23.45 ± 0.92	23.01 ± 0.99	0.58 ± 1.04	0.76 ± 1.34	0.89 ± 0.73	0.83 ± 0.64	0.90 ± 0.74	0.82 ± 0.66	0.61 ± 0.46	0.66 ± 0.41	0.38 ± 0.42	0.33 ± 0.33

TA: total astigmatism; CA: corneal astigmatism; IA: internal astigmatism; DIA: difference of internal astigmatism between both eyes. The axial length of boys was longer significantly than that of girls (*t* = 8.14, *P* < 0.001). A linear relationship between axial length and age was found in both genders (*t* = 10.56, *P* < 0.001 in male; *t* = 9.14, *P* < 0.001 in female). And there was no significant difference in IA and DIA between genders (*t* = 1.88, *P* > 0.05 for IA; *t* = 1.63, *P* > 0.05). IA and DIA did not grow significantly with age in both genders (*t* = 0.56, *P* > 0.05 in male, and *t* = 0.053, *P* > 0.05 in female, resp.).

**Table 2:** The axial length in different DIA.

Group	Cases	Axial length in the eye with less IA (mm)	Axial length in the eye with larger IA (mm)	t	*P*
DIA = 0 D	128	23.27 ± 0.99 (right eye)	23.29 ± 0.96 (left eye)	0.89	>0.05
0 < DIA< 0.5 D	292	23.22 ± 0.92	23.24 ± 0.95	0.44	>0.05
DIA ≥ 0.5 D	192	23.17 ± 0.95	23.28 ± 0.97	2.65	<0.05

DIA: difference of internal astigmatism between both eyes; IA: internal astigmatism.
